# Traditional Brazilian dietary pattern as a factor associated with lower prevalence of dynapenic abdominal obesity in hemodialysis patients

**DOI:** 10.1186/s12882-026-05032-7

**Published:** 2026-05-13

**Authors:** Monica Cattafesta, Luciane Bresciani Salaroli

**Affiliations:** 1 Postgraduate Program in Nutrition and Health, Federal University of Espírito Santo (UFES), Vitória, ES Brazil; 2 Postgraduate Program in Nutrition and Health and Postgraduate Program in Collective Health, Federal University of Espírito Santo (UFES), Vitória, ES Brazil

**Keywords:** Renal dialysis, Renal insufficiency chronic, Food consumption, Dietary patterns, Dynapenic abdominal obesity

## Abstract

**Background:**

Abdominal dynapenic obesity, defined by the combination of excessive abdominal fat accumulation and reduced muscle strength, is an important marker for assessing nutritional status in hemodialysis patients. However, evidence on how dietary patterns influence this condition is limited. Therefore, this study aimed to explore factors associated with adherence to dietary patterns in patients who require hemodialysis and assess the link between these patterns and dynapenic abdominal obesity.

**Methods:**

This is a cross-sectional study involving 996 patients requiring hemodialysis in Southeastern Brazil. Dietary patterns were identified using principal component analysis. Dynapenic abdominal obesity was defined by abdominal obesity and reduced muscle strength (measured by handgrip).

**Results:**

Overall, three dietary patterns were identified: ovovegetarian; cafeteria; and traditional Brazilian. Higher adherence was associated with approximately twofold higher odds among individuals with an income above two minimum wages and those who engaged in physical activity. Men, patients undergoing hemodialysis for a longer duration, those who engaged in physical activity, and alcohol consumers were more likely to adhere to the cafeteria pattern, whereas older patients requiring hemodialysis and those without paid employment were less likely to adhere to this pattern. Individuals with more education adopted the traditional Brazilian pattern less often, but men and exclusive users of the public health system adhered to it more commonly. After adjustments for confounding variables, patients requiring hemodialysis with greater adherence to the traditional dietary pattern had 40.4% lower odds of dynapenic abdominal obesity (OR = 0.596, 95% CI 0.435–0.816, *p* < 0.001).

**Conclusions:**

The findings underscore the complexity of dietary choices in patients undergoing hemodialysis, as these are influenced by a wide range of factors, and highlight that greater adherence to the traditional Brazilian dietary pattern was associated with lower odds of dynapenic abdominal obesity in this population.

**Clinical trial number:**

Not applicable.

**Supplementary Information:**

The online version contains supplementary material available at 10.1186/s12882-026-05032-7.

## Introduction

Non-communicable chronic diseases represent a serious public health issue, accounting for approximately 71% of deaths worldwide [1], many of which occur prematurely, and leading to disabilities and a decline in quality of life, thereby significantly burdens the healthcare system and social welfare [1, 2].

Among chronic diseases, kidney disorders are particularly concerning due to the continuous increase in the incidence and prevalence of chronic kidney disease (CKD), as well as the high costs across the care continuum [3]. It is estimated that 697.5 million people worldwide have CKD, representing 9.1% of the global population [4]. In Brazil, approximately 8%, nearly 17 million Brazilians, show some degree of kidney dysfunction [4]. The 2022 Brazilian Dialysis Census indicated that both the absolute number and the prevalence rate of patients undergoing chronic dialysis continue to rise in the country. In July 2022, there was a 3.7% increase in the number of patients undergoing chronic dialysis compared to 2021, with an estimated total of 153,831 patients (758 per million population) [5].

For these patients, renal replacement therapy options include various modalities, with hemodialysis being the most common, used by approximately 95.3% of patients with CKD in Brazil [5]. This method is recognized for its favorable prognoses and for significantly improving patient survival but its benefits are more pronounced among those who adhere to dietary, fluid, and medication recommendations [6].

The management of CKD is crucial for controlling the risk factors that accelerate its progression to kidney failure and for minimizing the risks associated with cardiovascular complications [2, 7, 8]. This requires patients to actively engage in disease self-management, which includes adhering to dietary recommendations, engaging in physical activity, following prescribed medications, managing symptoms, and seeking health information [2]. However, various challenges, such as inadequate dietary intake, hormonal and gastrointestinal disorders, dietary restrictions, polypharmacy, insufficient dialysis, uremia, and metabolic acidosis, can compromise the health of patients requiring dialysis, leading to nutrient loss, reduced functional capacity, poorer health outcomes, and increased mortality [2, 7, 8].

In developed countries, the nutritional transition process occurs rapidly as factors such as industrialization and urbanization drive adherence to Westernized dietary patterns, characterized by ultra-processed, hypercaloric foods with high levels of sugar, fats, sodium, and chemical additives, contributing to the rising incidence of CKD [9, 10]. Both CKD and obesity are considered ongoing clinical pandemics with significant impacts on public health and clinical care [11]. The relation between obesity and CKD is evident and frequently coexists in the same patient, with obesity playing a direct causal role in the onset, progression, and severity of CKD [11, 12]. Additionally, these patients often face dynapenia, a loss of muscle strength and power, creating a vicious cycle between abdominal obesity and dynapenia, which negatively impacts health and well-being, especially among the elderly, a phenomenon known as dynapenic abdominal obesity (DAO) [13, 14]. The presence of DAO is associated with various adverse outcomes, such as cognitive impairment, disability in instrumental activities of daily living, decline in physical performance, reduced gait speed, and increased risk of falls and fractures, and it may serve as a predictor of functional limitations and mortality in older adults [14]. In our studied population, 45.42% of individuals had DAO, highlighting the need for effective preventive and/or therapeutic interventions [15].

Despite the evidence linking dietary intake and nutritional status with the success of treatment in patients requiring hemodialysis, studies assessing adherence to dietary patterns in these patients, their associated factors, and the consequences for nutritional status remain scarce, especially considering *a posteriori* dietary patterns [16]. Despite the high consumption of ultra-processed foods among patients requiring hemodialysis [17], especially in those with abdominal obesity [18], the manner in which the dietary patterns of these patients may affect the coexistence of abdominal obesity and muscle strength loss is yet to be researched [15]. Therefore, this study aimed to explore the factors associated with adherence to dietary patterns among individuals undergoing hemodialysis and to evaluate the association between this adherence and the presence of DAO.

## Methods

### Study design

This analytical cross sectional study was conducted with users of hemodialysis services at Região Metropolitana da Grande Vitória, in Espírito Santo, Brazil.

### Sampling and data collection

Data were collected from February to September 2019 by inviting all individuals undergoing hemodialysis who attended all the 11 clinics in the region in 2019, which represented the totality of dialysis centers in the area during the study period. The target population of this study consisted of individuals undergoing hemodialysis of both sexes who were aged 18 years or above.

To meet the inclusion criteria, patients needed a confirmed CKD diagnosis in their medical records, to have undergone hemodialysis for at least six months, and be ambulatory. Individuals under contact precaution, those with ascites and those who were transferred to other clinics were excluded from this study.

As this was a population census (*n* = 1,351), all individuals who met the inclusion criteria were invited to participate in this study (*n* = 1,047). Of these, only 2.2% (*n* = 23) declined to participate, resulting in a total of 1,024 individuals initially included in this study (Figure S1).

### Independent variables

Based on the collected data, service users’ sociodemographic variables, lifestyle habits, and clinical history were analyzed. The sociodemographic variables included sex (“female” and “male”), age group (“19 to 29 years,” “30 to 59 years,” and “60 years or older”), marital status (“with partner” and “without partner”), education level (in years of schooling: “≤ 8 years,” “> 8 to ≤ 11 years,” and “> 11 years”), self-declared skin color (“white,” “black,” “brown,” “yellow,” “red”), family income (“≤ 2 minimum wages” and “> 2 minimum wages”), and employment status (“with paid employment,” including employed or retired/on medical leave, and “without paid employment,” with no personal income). Access to healthcare services was assessed with the question “How do you access healthcare services?” categorized as “mixed” (public and private), “public” (by the Brazilian Unified Health System), and “private” (health insurance and/or private). The evaluated clinical characteristics included time on hemodialysis replacement therapy (“< 6 years” and “≥ 6 years”), weekly frequency of hemodialysis (“< 3 times” and “≥ 3 times”), and average time spent on the hemodialysis equipment (“≤ 3 hours” and “> 3 hours”). The assessed lifestyle habits included self-reported alcohol consumption (“yes” and “no”), smoking (“non-smoker” and “current or former smoker”), and physical activity (“yes” and “no”).

### Dietary intake and dietary patterns

Dietary intake was assessed using the semi-quantitative Food Frequency Questionnaire (FFQ) from the ELSA-Brasil study, which has been validated for the Brazilian population [19, 20]. It is noteworthy that, to evaluate the internal consistency and reproducibility of the variables to be studied, a pilot study was conducted prior to data collection. Based on the pilot study data, a reliability analysis of the FFQ used was performed [19, 20], showing that all consumption frequencies of food items obtained substantial, almost perfect, or perfect agreement among their items. Additionally, the discrepancy between responses was low (0% to 14.3%), thus showing the applicability of this questionnaire in the studied population.

After collecting dietary data from the study population, the requirement of biological plausibility was observed [21]. Dietary data were collected for all 1,024 patients. Subsequently, the reported frequencies and quantities in the FFQ were converted into daily values [22] and nutrients were extracted using an evaluation template. After obtaining the nutrient data, 28 individuals (2.7%) were excluded due to implausible dietary intake, consisting of energy consumption ≤ 500 kcal or ≥ 6,000 kcal [21]. Thus, the final sample population comprised 996 individuals (Figure S1).

To define dietary patterns, Principal Component Analysis (PCA) was used. This methodology is recommended for estimating eating behavior that closely reflects the reality of a population [23, 24]. Initially, sample size adequacy was confirmed [24]. The internal consistency of the FFQ dimensions for the studied population was also evaluated and considered acceptable, with a Cronbach’s alpha = 0.604 [25]. Additionally, food items outside the habitual consumption of this group of individuals were excluded [26], namely: nuts, cashew nuts, Brazil nuts, peanuts, almonds, pistachios, beer, lentils, chickpeas, peas, wine, distilled alcoholic beverages (*cachaça*, whiskey, vodka), *acarajé*, and *chimarrão* (Table S1).

After identifying the frequently consumed food items, a correlation matrix was created for the remaining FFQ variables (69 food items), grouping them according to their nutritional characteristics and the correlation between food items [27] into 28 groups, as follows: Group 1: Rice; Group 2: Beans (black, red, white, string beans, etc.); Group 3: Garlic and onion; Group 4: Boiled potatoes, stewed potatoes, and mashed potatoes; Group 5: Polenta, cornmeal porridge, grits, and green corn; Group 6: Cassava/yuca, yam, taro, cooked plantain, and boiled sweet potatoes; Group 7: Lettuce; Group 8: Tomato; Group 9: Carrots, pumpkin/squash, cabbage, okra, sautéed kale and spinach, zucchini, chayote, eggplant, cauliflower, green beans, broccoli, beets, chicory, watercress, arugula, raw kale/endive/escarole/chard/raw spinach; Group 10: Banana; Group 11: Orange, mandarin, tangerine, pokan/bergamot; Group 12: Papaya, pineapple, grapes, mango, apple, pear, watermelon, and melon; Group 13: Eggs; Group 14: Pasta (cannelloni, lasagna, ravioli, tortelli); Group 15: Cassava flour, cornmeal, farofa, savory couscous, and paulista couscous; Group 16: Boneless beef (steak, ground beef, stewed beef), pork, tripe, and stewed tripe; Group 17: Chicken breast, Chester, turkey, cooked chicken, cooked fish/Capixaba stew, baked fish, stewed fish, grilled fish, and fried fish; Group 18: Sausages, chorizo, ham, mortadella, capicola, salami, pâté, etc.; Group 19: Milk, white cheeses (*Minas frescal*, ricotta, cottage, buffalo mozzarella), yellow cheeses (standard Minas, mozzarella, prato, cheddar, canastra cheese, processed cheese such as Polenghi, etc.), and yogurt; Group 20: Vegetable soup; Group 21: Oats, granola, bran, and other cereals; Group 22: Whole wheat, rye, and light bread (white or whole wheat); Group 23: Sweet biscuits, sweet bread, homemade bread, French bread, sliced bread, pita bread, toasted bread, simple cake (without filling), and salty crackers (like water crackers and others); Group 24: Margarine and vegetable spreads; Group 25: Baked savory snacks (esfihas, empanadas, baked pastries, etc.), pizza, stroganoff, and cheese bread; Group 26: Pudding, milk-based desserts, mousse, chocolate bars, bonbons, *brigadeiro*, *dulce de leche*, party sweets, and creamy ice cream; Group 27: Coffee; Group 28: Soft drinks and natural, artificial, and industrialized juices.

Next, the applicability of the PCA method was assessed using the Kaiser-Meyer-Olkin (KMO = 0.705) coefficient test and the Bartlett test of sphericity (with *p* < 0.001) [24]. After confirming the applicability of the method to the data, the number of retained factors was determined based on the following criteria: components with eigenvalues greater than 1.0, the Cattell scree plot, and the conceptual significance of the identified patterns. To determine dietary patterns, an initial analysis was conducted without setting the number of patterns. Then, after analyzing the Cattell scree plot, a second model was constructed, fixing the number of patterns to be retained. In total, three factors were extracted in the analysis based on the inflection point of the line in the Cattell plot [24, 25].

Following the aforementioned procedure, PCA was applied to the food groups, selecting the Varimax rotation to obtain uncorrelated factors [24, 25]. Food items or food groups with factor loadings above 0.3 were considered to have a strong association with the component, which provides better information for describing a dietary pattern, as adopted in previous studies [26, 27]. Food groups with factor loadings ≥ 0.30 on more than one factor were examined, and when cross-loadings occurred, they were assigned to the factor in which they presented the highest loading, taking into account the interpretability and conceptual coherence of the pattern. Finally, the patterns were named based on the interpretability and characteristics of the items retained in each factor [24].

### Dynapenic abdominal obesity (DAO)

Anthropometric assessment was performed after the hemodialysis session, selecting the side opposite to the venous access. All measurements were taken three times and the arithmetic mean of waist circumference, height, and handgrip strength was calculated. Of the 996 individuals participating in the study, 67 were unable to undergo anthropometric assessment. Therefore, the presence of DAO was evaluated in 929 individuals (Figure S1).

Waist circumference was measured with the participant standing, with their arms extended along their body and their feet together by using a non-elastic Sanny^®^ Medical tape (American Medical do Brasil LTDA ME, São Bernardo do Campo, São Paulo, Brazil) positioned at the midpoint between the lower edge of the costal margin and the iliac crest [28]. Height was measured with barefoot participants standing upright with their arms extended along their body and their gaze fixed on a point on the horizon [28], using a portable stadiometer with 2 mm precision by Sanny^®^ Medical (American Medical do Brasil LTDA ME, São Bernardo do Campo, São Paulo, Brazil). The waist-to-height ratio was obtained by dividing the waist circumference (cm) by height (cm) and was classified as indicating abdominal obesity when the value totaled ≥ 0.5 for both men and women [29].

Handgrip strength was assessed in participants’ dominant upper limb or in their hand without an arteriovenous fistula using a Saehan^®^ 5001 hydraulic hand dynamometer (Saehan Corp, Changwon-si, Gyeongsangnam-do, South Korea). Measurements were performed with participants seated in a chair without armrests with their elbow flexed at a 90° angle without resting it on their abdomen and with their feet flat on the floor. Overall, three measurements were taken with an average interval of 20 s, the mean value (kg) of which was used for analysis [30]. Muscle strength was classified using the cutoff point of 27 kg for men and 16 kg for women [31].

Thus, DAO was defined by combining dynapenia (muscle strength loss), measured by handgrip strength, and abdominal obesity, confirmed by the waist-to-height ratio [15].

### Statistical analysis

The normality of the variables was assessed by the Shapiro-Wilk test. To describe the study variables, the median (50p) was used as a measure of central tendency and the interquartile range (IQR) as a dispersion measure for continuous variables and absolute and percentage values ​​for categorical variables. Regarding the association tests between the independent variables and the outcome for the qualitative variables, the Pearson’s chi-squared test was used. When the expected values in the table cells remained below five or when the sum of the column value was less than 20, the Fisher’s exact test was used. Due to their abnormal distribution, the Mann-Whitney U test was used to analyze the association between a quantitative and a qualitative variable. Missing data were maintained due to low data loss, and varying numbers of individuals in each variable were reported in the table captions.

The binary logistic regression model was applied to assess the association between independent variables and adherence to dietary patterns (adherence below the median *versus* adherence above the median) and to assess the association between DAO and adherence to dietary patterns. Variables associated with dietary patterns and DAO indices at *p* < 0.05 in the association analyses were entered into the multivariable models (Table 2 and Table S2). Moreover, calorie consumption was used as an adjustment variable for these final models.

The Enter method was used for variable selection. The assumptions of binary logistic regression were assessed, including the absence of multicollinearity (tolerance > 0.1 and variance inflation factor < 10), adequate sample size relative to the number of variables included in the model (> 20 individuals per variable and > 5 cases per category), and the absence of influential outliers.

For all analyses, the level of significance adopted was α < 5%. They were performed using the statistical software IBM SPSS Statistics for Windows, version 24.0 (Armonk, NY: IBM Corp).

### Ethical statement

This research was approved by the Research Ethics Committee at Universidade Federal do Espírito Santo (Ufes) under protocol number 2.104.942 and CAAE 68528817.4.0000.5060. Participation was voluntary, and consent was obtained in writing by the signing of an informed consent form, following all the recommendations of the Declaration of Helsinki.

## Results

This study found three dietary patterns in the population of interest, namely: (1) Ovo-vegetarian, composed mainly of vegetables, fruits, tubers, cereals, and eggs; (2) Cafeteria diet, primarily composed of baked goods, sweets, meats, sweetened beverages, and coffee; and (3) Traditional Brazilian, characterized by the traditional Brazilian dish with rice, beans, seasonings such as onion and garlic, lettuce, and tomato (Table 1). It is noteworthy that the most consumed foods in this population include typical Brazilian foods, such as rice (96.4%), garlic (95.8%), beans (91.7%), coffee (91.2%), onions (89.3%), breads (88.8%), eggs (88.7%), lettuce (86%), pasta (85.2%), beef (82.6%), and tomatoes (81.3%) (Table S1).

**Table 1:** Distribution of factor loadings for food items/food groups across the three dietary patterns identified for patients requiring hemodialysis

Food Items	Dietary Patterns		
	1	2	3
	Ovovegetarian	Cafeteria	Traditional Brazilian
Chicory, watercress, arugula, kale, endive, escarole, chard, and raw spinach; sautéed kale and spinach; cabbage; pumpkin/squash; Italian zucchini; chayote and eggplant; green beans; okra; beets; cauliflower; broccoli; and carrots	**0.667**	−0.066	0.098
Lettuce	**0.566**	−0.034	**0.321**
Orange, mandarin, tangerine, and pokan/bergamot	**0.490**	0.204	−0.135
Tomato	**0.479**	0.025	**0.336**
Papaya, apple, and pear; pineapple; grapes; mango; watermelon; and melon	**0.478**	0.192	−0.224
Polenta, cornmeal porridge, grits, and corn	**0.385**	0.130	0.011
Oats, granola, bran, and other cereals	**0.376**	−0.051	−0.120
Fried, scrambled, boiled, poached eggs, and omelet	**0.352**	−0.008	0.177
Banana	**0.341**	0.201	−0.170
Cassava/yuca, yam, taro, plantain, and boiled sweet potatoes	**0.308**	0.236	−0.013
Baked savory snacks (esfihas, empanadas, baked pastries, etc.), stroganoff, cheese bread, and pizza	−0.004	**0.523**	−0.057
Sausages, chorizo, ham, mortadella, capicola, salami, pâté, etc.	−0.019	**0.498**	0.065
Regular, diet, and light soft drinks, and industrialized, artificial, and natural juices without sugar, with sugar, and with sweeteners	−0.001	**0.492**	−0.045
French bread, sliced bread, pita bread, toasted bread, simple cake (without filling), salty crackers (like water crackers and others), and sweet biscuits with and without filling	0.066	**0.467**	0.152
Coffee without sugar, with sugar, and with sweeteners	−0.066	**0.461**	0.187
Boneless beef (steak, ground beef, stewed beef), tripe/stewed tripe, and pork	0.136	**0.453**	0.014
Chocolate bars, bonbons, *brigadeiro*, *dulce de leche*, party sweets, pudding, milk-based desserts, mousse, and creamy ice cream	0.141	**0.413**	−0.190
Beans (black, red, white, string beans, etc.)	0.004	0.128	**0.702**
White and brown rice	0.000	0.228	**0.659**
Onion and garlic	0.011	−0.234	**0.585**
Margarine and vegetable spreads	−0.097	0.298	**0.307**
Boiled, stewed, or mashed potatoes	0.290	0.229	0.275
Pasta (cannelloni, lasagna, ravioli, tortelli)	0.154	0.263	0.135
Farofa, savory couscous, paulista couscous, cassava flour, and cornmeal	0.041	0.270	0.203
Chicken breast, Chester, turkey, etc.; cooked chicken (other parts); cooked fish/Capixaba stew; baked fish, stewed fish, grilled fish, and fried fish	0.253	0.073	0.154
Whole milk, skimmed milk, semi-skimmed milk, and plant-based milk; regular and light yogurt; white cheeses (Minas frescal, ricotta, cottage, buffalo mozzarella); and yellow cheeses (standard *Minas*, mozzarella, prato, cheddar, canastra, Polenghi-type, etc.)	0.226	0.159	0.145
Vegetable soup	0.266	−0.010	−0.014
Whole wheat or rye bread, and light bread (white or whole wheat)	0.261	−0.088	−0.024
*Explained individually variance (%)*	*8.961*	*7.910*	*7.200*
*Explained cumulative variance (%)*	*8.961*	*16.871*	*24.070*

Principal Component Analysis

The ovovegetarian dietary pattern was associated with age group, education level, skin color, family income, type of healthcare coverage, time on hemodialysis, and physical activity (Table 2). The cafeteria diet pattern was associated with sex, age group, employment status, time on hemodialysis, alcohol consumption, smoking, and physical activity. The traditional Brazilian pattern was associated with sex, education level, skin color, family income, and type of healthcare coverage.

**Table 2:** Association between sociodemographic variables, lifestyle habits, type of healthcare access, and clinical conditions with adherence to dietary patterns in patients undergoing renal replacement therapy on hemodialysis

Variables	Pattern 1 – Ovovegetarian			Pattern 2 - Cafeteria			Pattern 3 - Traditional Brazilian		
	Below the Median	Above the Median	*p*-value	Below the Median	Above the Median	*p*-value	Below the Median	Above the Median	*p*-value
	n (%)	n (%)		n (%)	n (%)		n (%)	n (%)	
**Sex** (*n* = 996)			0.848			**< 0.001**			**0.009**
Female	220 (50.5)	216 (49.5)		264 (60.6)	172 (39.4)		239 (54.8)	197 (45.2)	
Male	278 (49.6)	282 (50.4)		234 (41.8)	326 (58.2)		259 (46.3)	301 (53.8)	
**Age Group** (*n* = 996)			**0.018**			**< 0.001**			0.650
19 to 29 years	36 (63.2)^a^	21 (36.8)^a^		16 (28.1)^a^	41 (71.9)^a^		25 (43.9)	32 (56.1)	
30 to 59 years	267 (52.1)	245 (47.9)		225 (43.9)^b^	287 (56.1)^b^		257 (50.2)	255 (49.8)	
60 years or older	195 (45.7)^b^	232 (54.3)^b^		257 (60.2)^c^	170 (39.8)^c^		216 (50.6)	211 (49.4)	
**Marital Status** (*n* = 996)			0.655			0.225			0.566
With partner	274 (49.3)	282 (50.7)		288 (51.8)	268 (48.2)		283 (50.9)	273 (49.1)	
Without partner	224 (50.9)	216 (49.1)		210 (47.7)	230 (52.3)		215 (48.9)	225 (51.1)	
**Education Level** (*n* = 986)			**0.007**			0.153			**< 0.001**
≤ 8 years	265 (52.5)	240 (47.5)		266 (52.7)	239 (47.3)		221 (43.8)^a^	284 (56.2)^a^	
8 to ≤ 11 years	169 (52.0)	156 (48.0)		149 (45.8)	176 (54.2)		159 (48.9)^b^	166 (51.1)^b^	
11 years	60 (38.5)^a^	96 (61.5)^a^		76 (48.7)	80 (51.3)		110 (70.5)^c^	46 (29.5)^c^	
**Skin Color** (*n* = 983)			**0.004**			0.274			**0.027**
White	112 (41.8)^a^	156 (58.2)^a^		144 (53.7)	124 (46.3)		152 (56.7)^a^	116 (43.3)^a^	
Black	129 (55.6)	103 (44.4)		114 (49.1)	118 (50.9)		108 (46.6)	124 (53.4)	
Brown	251 (52.0)	232 (48.0)		230 (47.6)	253 (52.4)		229 (47.4)	254 (52.6)	
**Family Income** (*n* = 961)			**< 0.001**			0.105			**< 0.001**
≤ 2 minimum wages	308 (57.7)	226 (42.3)		277 (51.9)	257 (48.1)		235 (44.0)	299 (56.0)	
2 minimum wages	173 (40.5)	254 (59.5)		199 (46.6)	228 (53.4)		246 (57.6)	181 (42.4)	
**Employment Status** (*n* = 982)			0.092			**< 0.001**			0.737
With paid employment	154 (46.1)	180 (53.9)		136 (40.7)	198 (59.3)		165 (49.4)	169 (50.6)	
Without paid employment	336 (51.9)	312 (48.1)		358 (55.2)	290 (44.8)		328 (50.6)	320 (49.4)	
**Healthcare Coverage Type** (*n* = 995)			**< 0.001**			0.122			**< 0.001**
Mixed	10 (41.7)	14 (58.3)		7 (29.2)	17 (70.8)		19 (79.2)^a^	5 (20.8)^a^	
Public	407 (54.2)^a^	344 (45.8)^a^		380 (50.6)	371 (49.4)		338 (45.0)^b^	413 (55.0)^b^	
Private	80 (36.4)^b^	140 (63.6)^b^		111 (50.5)	109 (49.5)		140 (63.6)^c^	80 (36.4)^c^	
**Time on Hemodialysis** (*n* = 943)			**0.003**			**0.003**			0.541
< 6 years	286 (47.1)	321 (52.9)		333 (54.9)	274 (45.1)		297 (48.9)	310 (51.1)	
≥ 6 years	192 (57.1)	144 (42.9)		150 (44.6)	186 (55.4)		172 (51.2)	164 (48.8)	
**Weekly Frequency of Hemodialysis** (*n* = 996)			0.217*			0.687*			0.217*
< 3 times	1 (16.7)	5 (83.3)		4 (66.7)	2 (33.3)		5 (83.3)	1 (16.7)	
≥ 3 times	497 (50.2)	493 (49.8)		494 (49.9)	496 (50.1)		493 (49.8)	497 (50.2)	
**Average Time on Hemodialysis Machine** (*n* = 996)			0.056			0.832			0.290
≤ 3 h	59 (59.6)	40 (40.4)		51 (51.5)	48 (48.5)		55 (55.6)	44 (44.4)	
3 h	439 (48.9)	458 (51.1)		447 (49.8)	450 (50.2)		443 (49.4)	454 (50.6)	
**Alcohol Consumption** (*n* = 996)			0.823			**< 0.001**			0.823
No	453 (49.8)	456 (50.2)		475 (52.3)	434 (47.7)		453 (49.8)	456 (50.2)	
Yes	45 (51.7)	42 (48.3)		23 (26.4)	64 (73.6)		45 (51.7)	42 (48.3)	
**Smoking Status** (*n* = 996)			0.439			**0.002**			0.302
Non-smoker	301 (51.1)	288 (48.9)		319 (54.2)	270 (45.8)		303 (51.4)	286 (48.6)	
Current or former smoker	197 (48.4)	210 (51.6)		179 (44.0)	228 (56.0)		195 (47.9)	212 (52.1)	
**Physical Activity** (*n* = 975)			**0.007**			**< 0.001**			0.232
No	402 (51.7)	376 (48.3)		411 (52.8)	367 (47.2)		381 (49.0)	397 (51.0)	
Yes	80 (40.6)	117 (59.4)		75 (38.1)	122 (61.9)		106 (53.8)	91 (46.2)	

Pearson’s Chi-Squared Test. * Fisher’s Exact Test. Identical letters indicate differences between those categories when the variable has three or more categories

Higher adherence to the ovovegetarian dietary pattern remained associated with higher income and engagement in physical activity (Table 3). Patients requiring hemodialysis who earn more than two minimum wages (OR = 1.616, 95%CI = 1.173–2.227, *p* = 0.003) and those who engage in physical activity (OR = 1.614, 95%CI = 1.139–2.286, *p* = 0.007) were twice as likely to adhere more closely to this dietary pattern.

**Table 3:** Adjusted models for the association between sociodemographic variables, lifestyle habits, type of healthcare access, and clinical conditions with adherence to the ovovegetarian dietary pattern, cafeteria diet pattern, and traditional Brazilian pattern

Pattern 1 – Ovovegetarian*			
Variables	OR	95%CI	*p*-value
**Age Group** (*n* = 996)			
19 to 29 years	1		
30 to 59 years	1.673	(0.887–3.156)	0.112
60 years or older	1.787	(0.927–3.444)	0.083
**Education Level** (*n* = 986)			
≤ 8 years	1		
8 to ≤ 11 years	0.846	(0.607–1.180)	0.324
11 years	1.207	(0.764–1.906)	0.421
**Skin Color** (*n* = 983)			
White	1		
Black	0.683	(0.458–1.018)	0.061
Brown	0.761	(0.541–1.070)	0.116
**Family Income** (*n* = 961)			
≤ 2 minimum wages	1		
2 minimum wages	1.616	(1.173–2.227)	**0.003**
**Healthcare Coverage Type** (*n* = 995)			
Mixed	1		
Public	0.721	(0.288–1.801)	0.483
Private	1.065	(0.411–2.759)	0.897
**Time on Hemodialysis** (*n* = 943)			
< 6 years	1		
≥ 6 years	0.773	(0.577–1.036)	0.085
**Physical Activity** (*n* = 975)			
No	1		
Yes	1.614	(1.139–2.286)	**0.007**

Pattern 2 - Cafeteria**			
**Variables**	**OR**	**95%CI**	**p-value**
**Sex** (*n* = 996)			
Female	1		
Male	1.900	(1.422–2.540)	**< 0.001**
**Age Group** (*n* = 996)			
19 to 29 years	1		
30 to 59 years	0.510	(0.258–1.008)	0.053
60 years or older	0.288	(0.143–0.578)	**< 0.001**
**Employment Status** (*n* = 982)			
With paid employment	1		
Without paid employment	0.627	(0.462–0.850)	**0.003**
**Time on Hemodialysis** (*n* = 943)			
< 6 years	1		
≥ 6 years	1.554	(1.157–2.087)	**0.003**
**Alcohol Consumption** (*n* = 996)			
No	1		
Yes	2.036	(1.182–3.507)	**0.010**
**Smoking Status** (*n* = 996)			
Non-smoker	1		
Current or former smoker	1.641	(1.227–2.195)	**0.001**
**Physical Activity** (*n* = 975)			
No	1		
Yes	1.537	(1.081–2.184)	**0.017**

Pattern 3 - Traditional Brazilian***			
**Variables**	**OR**	**95%CI**	**p-value**
**Sex** (*n* = 996)			
Female	1		
Male	1.515	(1.155–1.987)	**0.003**
**Education Level** (*n* = 986)			
≤ 8 years	1		
8 to ≤ 11 years	0.887	(0.654–1.203)	0.440
11 years	0.461	(0.296–0.718)	**0.001**
**Skin Color** (*n* = 983)			
White	1		
Black	1.294	(0.883–1.895)	0.186
Brown	1.250	(0.902–1.732)	0.180
**Family Income** (*n* = 961)			
≤ 2 minimum wages	1		
2 minimum wages	0.791	(0.582–1.075)	0.134
**Healthcare Coverage Type** (*n* = 995)			
Mixed	1		
Public	3.589	(1.29–9.984)	**0.014**
Private	2.014	(0.701–5.787)	0.193

Binary logistic regression analysis. OR: odds ratio; 95%CI: 95% Confidence Interval. * Adjusted for age group, education level, skin color, family income, healthcare coverage type, time on hemodialysis, and physical activity. Hosmer-Lemeshow test: 0.624; ** Adjusted for sex, age group, employment status, time on hemodialysis, alcohol consumption, smoking, and physical activity. Hosmer-Lemeshow test: 0.953; *** Adjusted for sex, education level, skin color, family income, and healthcare coverage type. Hosmer-Lemeshow test: 0.997

Male individuals were almost twice as likely to adhere to the cafeteria diet pattern (OR = 1.900, 95%CI = 1.422–2.540, *p* < 0.001), whereas patients requiring hemodialysis aged 60 or older were 71.2% less likely to adhere to this dietary pattern (OR = 0.288, 95%CI = 0.143–0.578, *p* < 0.001). Similarly, those without paid employment at the time of data collection were 37.3% less likely to adhere to this dietary pattern (OR = 0.627, 95%CI = 0.462–0.850, *p* = 0.003). On the other hand, patients who had been on hemodialysis for a longer period (OR = 1.554, 95%CI = 1.157–2.087, *p* = 0.003), who consumed alcoholic beverages (OR = 2.036, 95%CI = 1.182–3.507, *p* = 0.010), who smoked (OR = 1.641, 95%CI = 1.227–2.195, *p* = 0.001), and who engaged in physical activity (OR = 1.537, 95%CI = 1.081–2.184, *p* = 0.017) were 1.5 to 2.0 times more likely to adhere to this less healthy dietary pattern.

Regarding greater adherence to the traditional Brazilian dietary pattern, patients requiring hemodialysis with more than eleven years of education were 54% less likely to adhere to this pattern (OR = 0.461, 95%CI = 0.296–0.718, *p* = 0.017), whereas men were 51% more likely (OR = 1.515, 95%CI = 1.155–1.987, *p* = 0.003). Additionally, those who accessed healthcare exclusively by the public system were more than three times more likely to adhere to the traditional Brazilian pattern (OR = 3.589, 95%CI = 1.29–9.984, *p* = 0.014).

This study also observed an association between adherence to dietary patterns and the presence of DAO, particularly with the cafeteria diet and traditional Brazilian patterns (*p* < 0.001) (Table 4).

**Table 4:** Dynapenic abdominal obesity (DAO) and adherence to dietary patterns in patients requiring hemodialysis

Dietary Patterns		Without DAO	With DAO	*p*-value
**Ovovegetarian**	[p50 (IQR)]	−0.23 (− 0.67. 0.38)	−0.27 (− 0.60. 0.31)	0.716*
Below the median	[n (%)]	191 (41.2)	273 (58.8)	0.525**
Above the median	[n (%)]	201 (43.2)	264 (56.8)	
**Cafeteria**	[p50 (IQR)]	0.03 (− 0.48. 0.67)	−0.34 (− 0.71. 0.24)	< 0.001*
Below the median	[n (%)]	148 (32.5)	308 (67.5)	< 0.001**
Above the median	[n (%)]	244 (51.6)	229 (48.4)	
**Traditional Brazilian**	[p50 (IQR)]	0.12 (− 0.64. 0.78)	−0.27 (− 0.82. 0.55)	< 0.001*
Below the median	[n (%)]	164 (35.3)	300 (64.7)	< 0.001**
Above the median	[n (%)]	228 (49.0)	237 (51.0)	

* Mann-Whitney U Test. ** Pearson’s Chi-Squared Test. *N* = 929. DAO: Dynapenic Abdominal Obesity; p50: 50th percentile, median; IQR: interquartile range (25th percentile – 75th percentile)

After adjustments, the traditional Brazilian pattern remained associated with DAO (Table 5), in which those with greater adherence to the traditional pattern had lower odds of DAO (OR = 0.596, 95%CI = 0.435–0.816, *p* < 0.001).

**Table 5:** Adjusted model of the association between dynapenic abdominal obesity and adherence to dietary patterns in patients requiring hemodialysis

Variables	DAO					
	Crude			Adjusted*		
	OR	95%CI	*p*-value	OR	95%CI	*p*-value
**Ovovegetarian**			0.525			0.166
Below the median	1			1		
Above the median	0.919	(0.708–1.192)		1.250	(0.912–1.714)	
**Cafeteria**			**< 0.001**			0.058
Below the median	1			1		
Above the median	0.451	(0.346–0.589)		0.716	(0.506–1.012)	
**Traditional Brazilian**			**< 0.001**			**0.001**
Below the median	1			1		
Above the median	0.568	(0.437–0.739)		0.596	(0.435–0.816)	

Binary logistic regression analysis. * Adjusted for caloric intake, sex, age group, education level, employment status, alcohol consumption, and physical activity. Hosmer-Lemeshow test: pattern 1 = 0.305, pattern 2 = 0.758, pattern 3 = 0.918. OR: odds ratio; 95%CI: 95% Confidence Interval

## Discussion

This study found three dietary patterns in this population of patients requiring hemodialysis: the “ovovegetarian” pattern, predominantly composed of vegetables, fruits, tubers, cereals, and eggs; the “cafeteria diet” pattern, characterized by a predominance of baked goods, sweets, meats, sweetened beverages, and coffee; and the “traditional Brazilian” pattern, marked by the consumption of rice, beans, seasonings such as onion and garlic, lettuce, and tomatoes. Using a similar *a* *posteriori* methodology, dietary consumption analyses identified three dietary patterns in Portuguese patients requiring hemodialysis: Mediterranean (traditional in the country), Western, and low in animal protein [32]. Another longitudinal study found a “prudent” dietary pattern and a “flour and meat” pattern; the prudent pattern was inversely associated with the risk of renal function decline in men [33]. A population from Western Asia exhibited lactovegetarian, high in fats and sugars, and traditional local (Iranian) patterns [34]. An *a* *priori* analysis of dietary consumption in Australian patients requiring hemodialysis indicated that diet quality was considered suboptimal, characterized by high intake of processed foods and animal protein and low intake of fruits and vegetables [2].

Despite the importance of studying dietary patterns rather than evaluating nutrient intake in isolation within this population [2], literature on *a posteriori* dietary patterns in patients requiring hemodialysis remains scarce [16, 32]. The qualitative approach to dietary consumption offers the advantage of reducing the focus on achieving “adequate” nutrient levels by emphasizing dietary factors related to food composition and consumption patterns, given that nutrient intake does not occur in isolation [35] and nutrients act synergistically in the body [36]. In this context, the World Health Organization recommends that dietary assessments of populations should be based on dietary patterns rather than isolated nutrients [37]. Dietary patterns are a valuable tool for reflecting eating behavior more closely aligned with population reality [23, 24] and for capturing the complex combination of nutrients and anti-nutritional factors involved in the human diet [23]. Furthermore, medical and nutritional recommendations for patients with CKD encourage the adoption of high-quality dietary patterns, characterized by high intake of fruits, vegetables, and whole grains and low intake of saturated fats, sodium, and refined sugars, in contrast to recommendations based solely on isolated nutrients, which can omit entire food groups and lower diet quality [2].

It is important to highlight that the dietary patterns identified in this population of patients with CKD resemble those of the general Brazilian population. The latter include a traditional pattern characterized by the consumption of rice and beans—although it also includes meats—a bread and butter/margarine pattern, characterized by the consumption of bread and oils and fats, and a Western pattern, which includes sodas, pizzas, and snacks [38]. This similarity may be partly attributed to the dietary recommendations patients receive regarding the restriction of animal proteins. In addition, this study observed that, apart from the traditional pattern, which does not account for he high consumption of meat, patients with renal impairments showed a vegetarian pattern. Notably, in the Brazilian dietary patterns identified in the general population, no group showed significant intake of fruits, vegetables, and greens. This discrepancy may be attributed to more targeted nutritional guidance provided to patients requiring hemodialysis, who receive support from a multidisciplinary team. Furthermore, such teams are mandatorily composed of nutritionists, as established by Collegiate Board Resolution No. 11, dated March 13, 2014, issued by the National Health Surveillance Agency [39]. This suggests that nutritional interventions by these professionals may offer significant benefits in improving diet quality in adults with CKD [40].

The labelling of Pattern 3 as “Traditional Brazilian” follows the methodological criterion established in the dietary pattern literature, whereby component names are assigned based on the foods with the highest factor loadings in each factor, interpreted in conjunction with the cultural characteristics of the population under study [24, 25]. Group 24 comprises industrially produced margarine and commercially manufactured vegetable fat spreads, which are classified as ultra-processed foods under the NOVA system owing to their formulation with additives such as emulsifiers, flavourings, and colourants, and to the industrial processes — hydrogenation or interesterification — involved in their production [41]. These products are therefore distinct from culinary preparations derived from fresh or minimally processed plant foods, such as homemade nut pastes or avocado-based spreads, which belong to different NOVA categories and carry substantially different nutritional profiles. The presence of margarine and vegetable spreads (Group 24, factor loading = 0.307) does not invalidate this characterisation; rather, it reflects the actual dietary behaviour of the Brazilian population as documented by the 2017–2018 Household Budget Survey (Pesquisa de Orçamentos Familiares – POF): French bread (*pão de sal*) was the fourth most frequently consumed food item in the country, reported by 50.9% of the population, and margarine was the single largest contributor to caloric intake among ultra-processed foods, accounting for 2.8% of total caloric intake, consistently associated with bread consumption particularly at breakfast and snack meals [42]. This combination therefore constitutes a widespread and culturally embedded dietary habit in Brazil, coexisting with the rice-and-beans core of the traditional diet, most markedly among lower-income households. Furthermore, as an *a* *posteriori* pattern recognition technique, PCA captures dietary structures as they empirically manifest in the population, without constraining them to normative pre-defined categories of healthy or unhealthy foods [24, 43]. A further analytical property of PCA warrants emphasis in this context: every individual in the sample receives a score on each identified pattern, meaning that participants are not classified into a single pattern but rather assessed according to their degree of adherence to each component [43]. The factor loading of margarine in this pattern therefore reflects its habitual co-variation with the other foods comprising this component in the everyday diet of the population, and the labels attributed to the patterns should be understood as interpretive descriptors rather than judgements of nutritional quality [24].

It is important to acknowledge that, although the factor loading of Group 24 (margarine and vegetable spreads, factor loading = 0.307) meets the conventional threshold for structural inclusion in the pattern, it represents the lowest loading among all items comprising Pattern 3, indicating a secondary rather than defining role in the co-variation structure captured by the analysis. Nevertheless, the pro-inflammatory potential of industrial margarine warrants explicit discussion. Ultra-processed fats, particularly those containing saturated and trans fatty acids, have been associated with elevated circulating concentrations of pro-inflammatory biomarkers, including C-reactive protein (CRP), interleukin-6 (IL-6), and tumour necrosis factor-alpha (TNF-α), through mechanisms involving gut microbiota dysbiosis, increased intestinal permeability, and direct activation of inflammatory signalling pathways [44]. However, the current evidence consistently indicates that the inflammatory effect of a dietary pattern is determined by its overall composition rather than by any single constituent food in isolation [44, 45]. The dominant items of Pattern 3 — beans, rice, and vegetables — possess well-documented anti-inflammatory and cardioprotective properties: legumes, in particular, are rich sources of dietary fibre, resistant starch, polyphenols, and bioactive peptides with demonstrated anti-inflammatory activity [46], and their regular consumption has been inversely associated with circulating inflammatory markers and cardiometabolic risk [47]. It is therefore plausible that the anti-inflammatory potential of the pattern’s core components substantially counterbalances the modest pro-inflammatory contribution of margarine at the consumption levels reflected by its factor loading — a hypothesis consistent with the broader literature demonstrating that the protective effects of traditional plant-rich dietary patterns operate through the synergistic action of their constituent foods as a whole [48]. It should be noted, however, that the present study did not directly assess inflammatory biomarkers, and the net inflammatory effect of Pattern 3 in this population therefore remains an open empirical question. Future research examining inflammatory biomarker profiles in relation to dietary pattern scores in Brazilian populations would contribute meaningfully to clarifying this interaction.

The protective potential of Pattern 3 may also extend to more specific biological mechanisms relevant to chronic kidney disease management. The high fibre content provided by beans, rice, and vegetables supports the production of short-chain fatty acids by colonic microbiota, which maintain intestinal epithelial integrity and attenuate systemic inflammation, while simultaneously limiting the generation of protein-bound uremic toxins — such as indoxyl sulfate and p-cresyl sulfate — that are implicated in CKD progression [49, 50]. Additionally, the potassium supplied by these plant foods has been associated with improved blood pressure control and slower CKD progression, with emerging evidence suggesting that the lower bioavailability of potassium from plant sources may mitigate hyperkalemia risk even in patients with reduced kidney function [51]. These pathways highlight the gut–kidney axis as a relevant mechanistic framework through which adherence to a traditional plant-rich dietary pattern may confer renal protection.

This scenario prompts a discussion of the factors associated with adherence to the identified dietary patterns. Adherence to the ovovegetarian pattern was higher among individuals with higher income, women, and those who engaged in physical activity. Although fruit and vegetable consumption is low across the Brazilian population as a whole, with an average of less than two fruits per day [52] — as also observed in this study — women and individuals from higher socioeconomic classes tend to have healthier eating habits [42, 53]. In contrast, men, younger individuals, and consumers of alcohol and tobacco, showed higher adherence to the cafeteria pattern, which was considered the least healthy of the three. In the Brazilian population, diet quality improves with age [53], and processed food consumption is more prevalent among younger individuals [54, 55]. The intake of ultra-processed foods in Brazil increased by 1.02% points (pp) from 2008 to 2009 to 2017–2018, this increase being more pronounced in men (+ 1.59 pp), among those with up to four years of education (+ 1.18 pp), and in the lowest income quintile (+ 3.54 pp) [9]. Popkin and Adair [56] argue that dietary changes: younger generations tend to adopting new dietary patterns more rapidly, while older individuals continue to eat in more traditional — and sometimes healthier — ways. This may also reflect the tendency of older individuals to modify their eating habits in response to the onset of chronic disease, leading them to selecting more nutritious foods [57].

In this context, the cafeteria dietary pattern also includes sugar-sweetened beverages. Despite a general reduction in soda consumption in Brazil, sodas ranked fifth among the foods with the highest daily per capita consumption averages, after coffee, beans, rice, and juices. However, this reduction was considerably smaller among Brazilians in the lowest income quartile than among those in the highest income quartile [42].

The association between physical activity and the ovovegetarian dietary pattern, and between alcohol and tobacco consumption and the cafeteria pattern, suggests that individuals who consume alcohol and tobacco, as well as sedentary individuals, tend to exhibit poorer dietary habits, reflecting the clustering of health-compromising behaviors [58]. Moreover, the consumption of baked goods and snacks, along with longer time on hemodialysis, may be related to the hemodialysis process itself. A study comparing chronic patients requiring hemodialysis with non-hemodialysis older adults found that the hemodialysis group had poorer dietary quality and higher consumption of processed and ultra-processed foods relative to the non-hemodialysis group. Furthermore, these patients showed poorer dietary quality specifically on dialysis days [59]. The authors suggest that changes in daily routines imposed by dialysis treatment, combined with limited access to healthier foods, may promote the substitution of vegetables, legumes, fruits, and other minimally processed foods with ultra-processed alternatives such as snacks, sandwiches, fast foods, and cookies. In addition, many dietary restrictions recommended for patients requiring hemodialysis may also contribute to the findings of this study. Specifically, dialysis patients are often advised to limit their intake of fruits, vegetables, and legumes to manage serum potassium and phosphorus levels [59]. Consequently, patients undergoing hemodialysis for a longer period may show greater adherence to the cafeteria dietary pattern as a result of compliance with more outdated recommendations that restrict the consumption of fruits and vegetables [16]. It is important to note, however, that such multiple dietary restrictions may compromise overall diet quality by reducing intake of fiber, vitamins, and essential nutrients, ultimately diverging from what is generally recommended as a healthy diet [59].

Finally, patients requiring hemodialysis without paid employment showed lower adherence to the cafeteria dietary pattern. Beyond ultra-processed foods — which remain relatively expensive in Brazil [52] — this pattern also includes meat products. It is noteworthy that Brazilians are among the highest consumers of beef in the world, accounting for 12% of global consumption [60]. This consumption exceeds the limits recommended by the World Health Organization, which sets a maximum of 25 kg per capita for all types of meat and 15 kg per capita for beef [61]. Nevertheless, meat consumption tends to be higher among Brazilians with higher incomes, reflecting its relatively high cost [62].

The traditional dietary pattern was associated with socioeconomic proxy variables, with greater adherence observed among individuals with lower educational levels, men, and those who exclusively use the public healthcare system. National data indicate that individuals with lower purchasing power tend to consume more foods characteristic of the traditional diet, with those earning up to two minimum wages per capita showing higher regular consumption of beans [53]. Similarly, men are the primary consumers of traditional foods such as rice and beans [42].

When analyzing how these dietary patterns might be related to the presence of DAO, the traditional dietary pattern remained associated. Patients on hemodialysis who adhered more to this pattern had approximately 40% lower odds of presenting DAO. This association may be related to the overall composition of the pattern, which is characterized by the consumption of cereals and legumes, home-prepared meals, and fresh ingredients. Such components have been consistently associated in the literature with more favorable metabolic and inflammatory profiles, as well as with a lower risk of obesity and muscle loss, which may partly explain the observed relationship between the traditional dietary pattern and DAO [63].

It should be noted that dynapenia, characterized by a decline in muscle strength and power [13], may be accompanied by an increase in abdominal fat mass attributable to aging, a lower metabolic rate, or myokine deficiency [12]. Conversely, abdominal obesity is associated with accelerated fat infiltration into skeletal muscle and inflammatory responses, both of which may contribute to decreased muscle strength and physical functionality [64]. This bidirectional relationship may give rise to a potentially vicious cycle between dynapenia and abdominal obesity, with consequent synergistic negative impacts on health and well-being, especially among older adults [14]. Furthermore, dynapenia, obesity, and CKD are progressive conditions that often co-occur and carry significant implications for public health and clinical practice [11].

Understanding these pathological processes highlights the potential relevance of dietary patterns in the context of CKD management, particularly with regard to the selection of dietary patterns that may positively influence patients’ nutritional status, metabolic profile, and inflammatory markers. Regarding the patterns identified in this study, both the ovovegetarian and cafeteria patterns showed no association with dynapenia, possibly reflecting their overall low nutrient density, low content of complete amino acids, and high levels of chemical additives, animal protein, fat, and sodium. It is important to note that plant-based diets differ from specific types of vegetarian diets (pescatarian, lacto-ovo-vegetarian, lacto-vegetarian, ovovegetarian, vegan, and others); the latter do not necessarily consider food quality or restrict refined and processed foods, unlike dietary patterns such as the DASH diet (Dietary Approaches to Stop Hypertension) and the Mediterranean diet [65]. Although no formal definition of a “plant-based” diet exists, this term is commonly used to refer to dietary patterns that promote the consumption of plant foods, including fruits, vegetables, whole grains, and plant proteins such as nuts, seeds, and legumes. Plant-based diets do not necessarily imply the complete exclusion of animal products. For example, the DASH and Mediterranean dietary patterns may be considered plant-based, despite not recommending the complete exclusion of meats and other animal products [66], as is also the case with the traditional pattern identified in this study, which is rich in cereals, legumes, natural seasonings, and vegetables.

In recent years, interest in plant-based diets as a strategy to slow the progression of CKD and manage associated comorbidities has increased significantly. A previous study has suggested that plant-based dietary patterns are associated with improvements in microalbuminuria, a slower decline in renal function, and reduced mortality risk in individuals with CKD [16]. Such diets have also proven beneficial for managing common comorbidities such as cardiovascular disease, hypertension, diabetes, and obesity [16, 65–67]. One important characteristic of plant-based dietary patterns is their lower dietary acid load. Diets high in animal protein tend to increase the acid load, thereby creating an environment conducive to the generation of reactive oxygen species and glomerular hyperfiltration injury [68, 69], which may significantly contributes to disease progression [65, 70].

Metabolic acidosis, resulting from an increased acid load, is associated with increased urinary acid excretion, bone resorption, and muscle catabolism — mechanisms that serve as compensatory responses to maintain serum pH and bicarbonate levels. Over time, these processes culminate in CKD progression, bone mineral disease, and sarcopenia [65]. Consequently, traditional diets based on a mix of grains, cereals, and vegetables have been associated with more favorable acid-base balance [68, 69]. While foods containing animal protein, such as meats, cheeses, and eggs, carry a positive potential renal acid load, certain plant proteins — such as those found in fruits, vegetables, and legumes, particularly beans — exhibit a negative potential renal acid load, which is advantageous for patients with CKD [68].

In addition to these benefits, the traditional Brazilian dietary pattern may be associated with characteristics that are potentially advantageous for renal health by reducing dietary phosphorus bioavailability and increasing dietary fiber intake [65]. Phosphorus in plant foods is less readily absorbed, whereas phosphorus from processed foods, due to the presence of chemical additives, may be considerably more bioavailable than that from plant-based sources [71]. Given the lack of the enzyme phytase, humans are unable to hydrolyse the phytic acid present in plants, resulting in intestinal phosphorus absorption of less than 40%, compared to an absorption rate of 40–60% for organic phosphates in animal products [65, 68]. Similarly, the bioavailability of potassium from plant sources is as low as 50–60%, compared to that of a diet richer in animal products (90% bioavailable) and chemical additives (90–100% bioavailable) [68, 72]. Several mechanisms are thought to account for this reduced bioavailability: reduced intestinal absorption attributable to plant cell walls; increased fecal excretion resulting from concurrent fiber consumption; greater potassium uptake into cells driven by concurrent bicarbonate and carbohydrate intake; and the influence of food preparation methods [65, 72].

It is also important to note that the updated guidelines of the National Kidney Foundation’s Kidney Disease Outcomes Quality Initiative have revised their recommendations regarding phosphorus and potassium intake, shifting towards an individualised approach that prioritises the bioavailability of these nutrients over total intake [73]. This suggests that diets with higher proportions of phosphorus and potassium derived from plant sources — such as cereals, grains, tubers, roots, fruits, and vegetables — may represent a viable and healthy alternative in the management of patients undergoing hemodialysis.

Finally, traditional dietary patterns, rich in whole grains and cereals, also provide a naturally high fiber diet, which is known for its positive effects on cardiovascular and gastrointestinal health [65, 74, 75]. High dietary fiber intake has been shown to significantly reduce serum urea and creatinine levels in patients with CKD. This effect is mediated by alterations in the composition of the intestinal microbiota, which promotes bacterial nitrogen capture in the colon. By reducing colonic transit time, dietary fibre decreases the renal burden associated with serum urea homeostasis, thereby facilitating greater intestinal excretion of nitrogenous waste [75].

Thus, diet quality is an important component of self-care in CKD management [2]. However, for self-care to be truly effective, it must be complemented by dietary and lifestyle modifications alongside other medical and pharmacological treatments. Diets centred on the consumption of minimally processed and unprocessed foods, especially those of a plant-based nature, have shown significant benefits for patients with CKD, while also presenting challenges that require careful consideration. Accordingly, empowering patients with the knowledge and resources to adopt a diet appropriate to their individual context and disease stage, supported by access to qualified nutritionists, is crucial for optimising health outcomes and ensuring a truly integrated therapeutic approach [76].

The use of the food frequency questionnaire represents an important limitation of this study, as it relies on a predefined list of foods and is subject to recall bias and measurement error. In patients undergoing hemodialysis, this limitation may be particularly relevant, since dietary intake is often influenced by restrictive renal diets, which can lead to systematic underreporting or misreporting of certain food groups. Additionally, the absence of a complementary dietary assessment method, such as a 24-hour dietary recall, precluded calibration of the instrument and may have affected the accuracy of intake estimation. Nevertheless, the rigorous pilot testing process and the assessment of reproducibility of this validated FFQ in the Brazilian population may have partially mitigated these sources of bias. Furthermore, the possibility of reverse causality, inherent to the cross-sectional study design, raises concerns that some important associations may have evaded identification, especially since major dietary changes tend to occur among younger individuals, whereas more severely ill and older patients may have recently changed their eating habits. Additionally, due to the cross-sectional design, temporality cannot be established. It is possible that patients with worse clinical status, abdominal obesity, or reduced muscle strength had already modified their dietary patterns following medical or nutritional advice, which may partially explain the observed associations. Nevertheless, this study stands out for addressing a significant gap in the literature, employing a methodology that is underexplored in this population group. Moreover, by evaluating not only obesity but also muscle loss, this study sheds light on a serious health issue that affects disease prognosis and related healthcare costs in this population.

## Conclusions

This study found three dietary patterns: ovovegetarian, cafeteria, and the traditional Brazilian. Adherence to the ovovegetarian pattern was associated with approximately twofold higher odds among individuals with an income above two minimum wages and those who engaged in physical activity. Men, patients undergoing hemodialysis for a longer duration, those who engaged in physical activity, and alcohol and tobacco users were more likely to adhere to the cafeteria pattern, whereas older patients on hemodialysis and those without paid employment were less likely to adhere to this pattern. Individuals with more than eight years of schooling were less likely to adopt the traditional Brazilian pattern, while men and exclusive users of the public health system adhered to it more frequently. After adjusting for confounding variables, patients who adhered most strongly to the traditional dietary pattern had 40.4% lower odds of DAO. The foods with the highest factor loadings in this pattern included beans, rice, onions, garlic, tomatoes, and lettuce.

Collectively, the findings of this study highlight the complexity of dietary choices in patients undergoing hemodialysis, which are influenced by a wide range of socioeconomic, behavioural, and clinical factors, and underscore that adherence to the traditional Brazilian dietary pattern was associated with lower odds of abdominal obesity in this population. These results point to the need for individualised nutritional approaches that consider these contextual factors to promote effective adherence to healthy dietary patterns. Furthermore, they suggest that adherence to a diet rich in the fundamental components of traditional traditional cuisine may represent a promising nutritional strategy for patients with CKD on hemodialysis. Nevertheless, these findings should be interpreted with caution, as further longitudinal and interventional studies are needed to corroborate them.

## Supplementary Information

Below is the link to the electronic supplementary material.

Supplementary Material 1

Supplementary Material 2

Supplementary Material 3

## Data Availability

The datasets generated and/or analyzed during the current study are not publicly available due to other ongoing studies using the same database, which need to be protected until their publication, but are available from the corresponding author on reasonable request.

## Abbreviations

50p: Median
95%CI: 95% Confidence Interval
CKD: Chronic kidney disease
DAO: Dynapenic abdominal obesity
DASH: Dietary Approaches to Stop Hypertension
FFQ: Food Frequency Questionnaire
IQR: Interquartile range
KMO: Kaiser-Meyer-Olkin
OR: Odds ratio
PCA: Principal Component Analysis
