# Supplementary material for: Traditional Brazilian dietary pattern as a factor associated with lower prevalence of dynapenic abdominal obesity in hemodialysis patients
Source: BMC Nephrol. 2026 May 13;27:409. doi: 10.1186/s12882-026-05032-7 (PMC13343747; doi:10.1186/s12882-026-05032-7)
Supplement: Supplementary file 1 — Supplementary Material 1 [file 12882_2026_5032_MOESM1_ESM.docx]

**Table Additional 1**. Frequency of Consumption of Food Items in the Population who Require Hemodialysis (%)

| **Food Item** | **% Consumption** | |
| --- | --- | --- |
| Rice | | 96.4 |
| Garlic | | 95.8 |
| Beans (black, red, white, string beans, etc.) | | 91.7 |
| Coffee | | 91.2 |
| Onion | | 89.3 |
| French bread/sliced bread/pita bread/toasted bread | | 88.8 |
| Eggs | | 88.7 |
| Lettuce | | 86.0 |
| Pasta (cannelloni, lasagna, ravioli, tortelli) | | 85.2 |
| Boneless beef (steak, ground beef, stewed beef) | | 82.6 |
| Tomato | | 81.3 |
| Chicken breast/Chester/turkey, etc. | | 79.2 |
| Boiled potatoes/stewed potatoes/mashed potatoes | | 77.7 |
| Cooked chicken (other parts) | | 75.1 |
| Polenta/cornmeal porridge/grits | | 74.6 |
| Cassava (yuca)/yam/taro, cooked plantain/boiled sweet potatoes | | 72.9 |
| Carrots | | 71.5 |
| Salty crackers (such as water crackers and others) | | 70.1 |
| Natural juice | | 70.0 |
| Milk | | 69.3 |
| Pumpkin (squash) | | 69.0 |
| Cabbage | | 68.6 |
| Okra | | 67.1 |
| Sautéed kale/spinach | | 64.3 |
| Apple/pear | | 63.7 |
| Zucchini/chayote/eggplant | | 62.8 |
| Cooked fish (Capixaba stew)/baked fish/stewed fish/grilled fish | | 62.7 |
| Pork | | 60.5 |
| Simple cake (without filling) | | 58.5 |
| Banana | | 58.3 |
| White cheeses (*Minas frescal*/ricotta/cottage/buffalo mozzarella) | | 56.9 |
| Margarine/vegetable spreads | | 56.5 |
| Orange/mandarin/tangerine/Pokan (bergamot) | | 55.7 |
| Cassava flour/cornmeal | | 52.8 |
| Papaya | | 52.6 |
| Cauliflower | | 52.3 |
| Fried fish | | 51.7 |
| Pineapple | | 49.2 |
| Baked savory snacks (esfiha/empanada/baked pastries, etc.) | | 46.5 |
| Sausages/chorizo (*salsichão*) | | 45.9 |
| Green beans | | 44.3 |
| Cheese bread | | 44.2 |
| Broccoli | | 43.9 |
| Grapes | | 43.8 |
| Beets | | 42.1 |
| Vegetable soup | | 41.6 |
| Mango | | 41.1 |
| Farofa/savory couscous/paulista couscous | | 40.9 |
| Pizza | | 39.8 |
| Sweet bread/homemade bread | | 39.7 |
| Yellow cheeses (standard Minas/mozzarella/prato/cheddar/canastra/processed like Polenghi, etc.) | | 38.3 |
| Yogurt | | 38.0 |
| Stroganoff | | 36.9 |
| Chicory/watercress/arugula/raw kale/endive/escarole/chard/raw spinach | | 35.8 |
| Ham/mortadella/capicola/salami/pâté, etc. | | 35.6 |
| Soft drinks | | 35.5 |
| Watermelon | | 35.4 |
| Green corn | | 33.7 |
| Creamy ice cream | | 33.1 |
| Sweet biscuits | | 32.9 |
| Tripe/stewed tripe | | 30.5 |
| Chocolate bars/bonbons, *brigadeiro*, *dulce de leche*/party sweets | | 29.1 |
| Pudding/milk-based desserts/mousse | | 26.0 |
| Melon | | 25.6 |
| Oats/granola/bran/other cereals | | 24.4 |
| Whole wheat/rye bread | | 16.6 |
| Industrialized juice | | 16.4 |
| Artificial juice | | 14.9 |
| Light bread (white or whole wheat) | | 11.6 |
| Nuts/cashew nuts/Brazil nuts/peanuts/almonds/pistachios | | 9.6 |
| Beer | | 8.7 |
| Lentils/chickpeas/peas | | 8.5 |
| Wine | | 7.4 |
| Distilled alcoholic beverages (cachaça, whiskey, vodka) | | 3.2 |
| *Acarajé* | | 2.1 |
| *Chimarrão* | | 0.2 |

Evaluated according to consumption frequency (yes versus no) relative to the total n (n = 996).
