# Supplementary material for: Traditional Brazilian dietary pattern as a factor associated with lower prevalence of dynapenic abdominal obesity in hemodialysis patients
Source: BMC Nephrol. 2026 May 13;27:409. doi: 10.1186/s12882-026-05032-7 (PMC13343747; doi:10.1186/s12882-026-05032-7)
Supplement: Supplementary file 2 — Supplementary Material 2 [file 12882_2026_5032_MOESM2_ESM.docx]

**Table Additional 2**. Analysis of Factors Associated with Dynapenic Abdominal Obesity to Choose the Adjustment Variables of the Multiple Models with Adherence to Dietary Patterns

| **Variables** | **Dynapenic Abdominal Obesity (DAO)** | | **p-value** |
| --- | --- | --- | --- |
|  | **Without DAO** | **With DAO** |  |
|  | **n (%)** | **n (%)** |  |
| **Sex** (n = 929) |  |  | **<0.001** |
| Female | 103 (25.20) | 305 (74.80) |  |
| Male | 289 (55.50) | 232 (44.50) |  |
| **Age Group** (n = 929) |  |  | **<0.001** |
| 19 to 29 years | 43 (76.80) | 13 (23.20) |  |
| 30 to 59 years | 252 (51.50) | 237 (48.50) |  |
| 60 years or older | 97 (25.30) | 287 (74.70) |  |
| **Marital Status** (n = 929) |  |  | 0.104 |
| With partner | 232 (44.50) | 289 (55.50) |  |
| Without partner | 160 (39.20) | 248 (60.80) |  |
| **Education Level** (n = 922) |  |  | **<0.001** |
| ≤ 8 years | 162 (34.10) | 313 (65.90) |  |
| 8 to ≤ 11 years | 161 (53.00) | 143 (47.00) |  |
| 11 years | 69 (48.30) | 74 (51.70) |  |
| **Skin Color** (n = 917) |  |  | 0.936 |
| White | 101 (41.20) | 144 (58.80) |  |
| Black | 93 (42.70) | 125 (57.30) |  |
| Brown | 193 (42.50) | 261 (57.50) |  |
| **Family Income** (n = 897) |  |  | 0.434 |
| ≤ 2 minimum wages | 207 (41.60) | 290 (58.40) |  |
| 2 minimum wages | 177 (44.30) | 223 (55.80) |  |
| **Employment Status** (n = 917) |  |  | **<0.001** |
| With paid employment | 178 (55.60) | 142 (44.40) |  |
| Without paid employment | 208 (34.80) | 389 (65.20) |  |
| **Healthcare Coverage Type** (n = 928) |  |  | 0.712 |
| Mixed | 9 (37.50) | 15 (62.50) |  |
| Public | 292 (41.70) | 409 (58.30) |  |
| Private | 90 (44.30) | 113 (55.70) |  |
| **Time on Hemodialysis** (n = 880) |  |  | 0.524 |
| < 6 years | 238 (41.80) | 331 (58.20) |  |
| ≥ 6 years | 137 (44.10) | 174 (55.90) |  |
| **Weekly Frequency of Hemodialysis** (n = 929) |  |  | 0.999 |
| < 3 times | 390 (42.20) | 534 (57.80) |  |
| ≥ 3 times | 2 (40.00) | 3 (60.00) |  |
| **Average Time on Hemodialysis Machine** (n = 929) |  |  | 0.180 |
| ≤ 3 hours | 32 (35.60) | 58 (64.40) |  |
| 3 hours | 360 (42.90) | 479 (57.10) |  |
| **Alcohol Consumption** (n = 929) |  |  | **<0.001** |
| No | 344 (40.50) | 506 (59.50) |  |
| Yes | 48 (60.80) | 31 (39.20) |  |
| **Smoking Status** (n = 929) |  |  | 0.687 |
| Non-smoker | 238 (42.70) | 319 (57.30) |  |
| Current or former smoker | 154 (41.40) | 218 (58.60) |  |
| **Physical Activity** (n = 909) |  |  | **0.005** |
| No | 284 (39.70) | 431 (60.30) |  |
| Yes | 99 (51.00) | 95 (49.00) |  |

Pearson’s chi-squared test. Variables selected for adjustment in the binary logistic regression analysis model: sex, age group, education, employment, alcohol consumption, and physical activity.
