# Supplementary figures and images for: Traditional Brazilian dietary pattern as a factor associated with lower prevalence of dynapenic abdominal obesity in hemodialysis patients
Source: BMC Nephrol. 2026 May 13;27:409. doi: 10.1186/s12882-026-05032-7 (PMC13343747; doi:10.1186/s12882-026-05032-7)

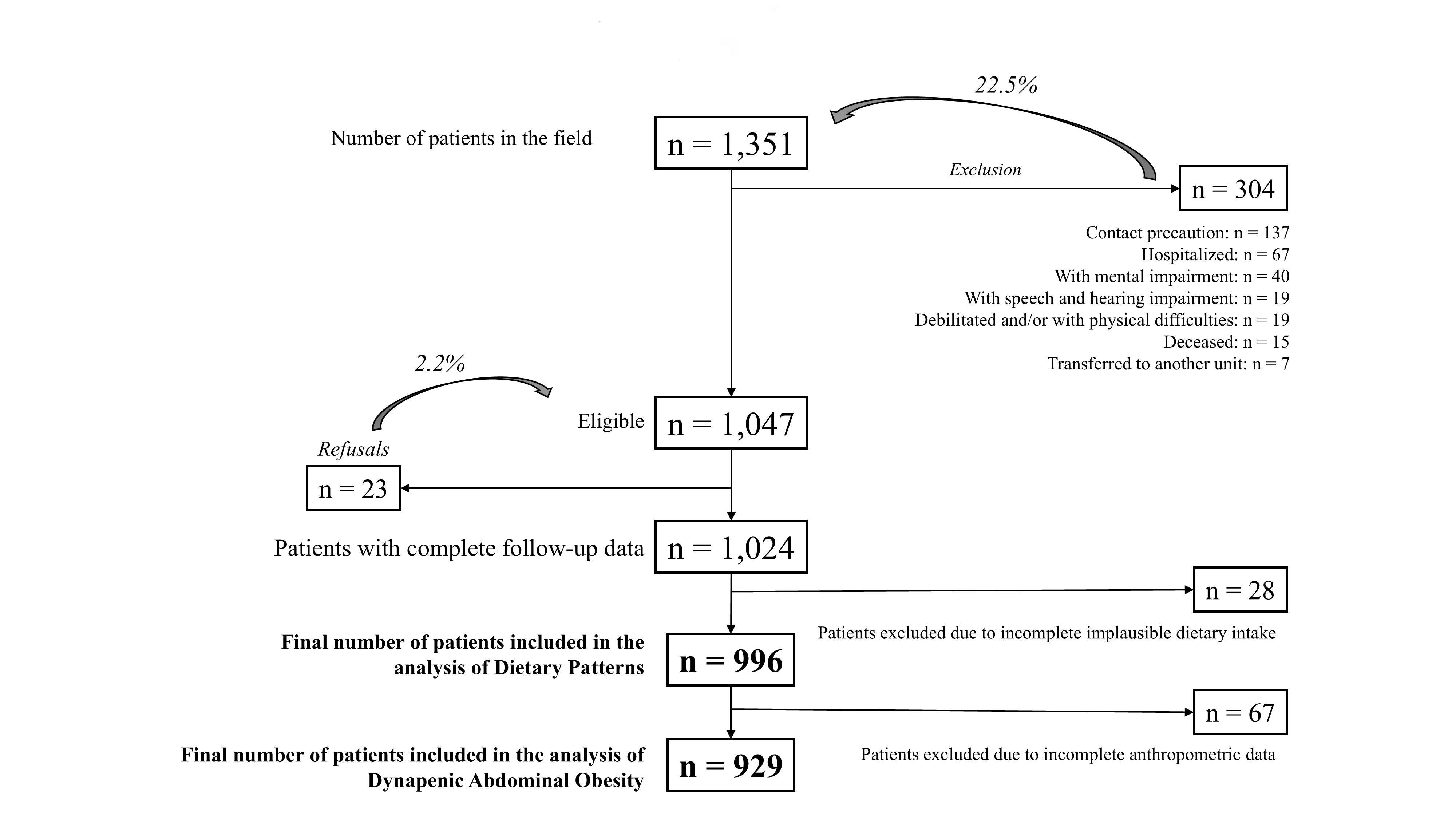

Supplement: Supplementary file 3 — Supplementary Material 3 [file 12882_2026_5032_MOESM3_ESM.tif]
